# Development of a Peanut Canopy Measurement System Using a Ground-Based LiDAR Sensor

**DOI:** 10.3389/fpls.2019.00203

**Published:** 2019-02-28

**Authors:** Hongbo Yuan, Rebecca S. Bennett, Ning Wang, Kelly D. Chamberlin

**Affiliations:** ^1^College of Mechanical and Electrical Engineering, Hebei Agricultural University, Baoding, China; ^2^Department of Biosystems and Agricultural Engineering, Oklahoma State University, Stillwater, OK, United States; ^3^USDA-ARS, Wheat, Peanuts and Other Field Crops Research Unit, Stillwater, OK, United States

**Keywords:** peanut cultivar, canopy height and density, image processing, classification, region of interest (ROI)

## Abstract

Plant architecture characteristics contribute significantly to the microclimate within peanut canopies, affecting weed suppression as well as incidence and severity of foliar and soil-borne diseases. However, plant canopy architecture is difficult to measure and describe quantitatively. In this study, a ground-based LiDAR sensor was used to scan rows of peanut plants in the field, and a data processing and analysis algorithm was developed to extract feature indices to describe the peanut canopy architecture. A data acquisition platform was constructed to carry the ground-based LiDAR and an RGB camera during field tests. An experimental field was established with three peanut cultivars at Oklahoma State University's Caddo Research Station in Fort Cobb, OK in May and the data collections were conducted once each month from July to September 2015. The ground-based LiDAR used for this research was a line-scan laser scanner with a scan-angle of 100°, an angle resolution of 0.25°, and a scanning speed of 53 ms. The collected line-scanned data were processed using the developed image processing algorithm. The canopy height, width, and shape/density were evaluated. Euler number, entropy, cluster count, and mean number of connected objects were extracted from the image and used to describe the shape of the peanut canopies. The three peanut cultivars were then classified using the shape features and indices. A high correlation was also observed between the LiDAR and ground-truth measurements for plant height. This approach should be useful for phenotyping peanut germplasm for canopy architecture.

## Introduction

Peanut (*Arachis hypogaea* L.) is a major crop which is widely cultivated in warmer areas of the United States and around the world. In the U.S., it is the 12th most valuable cash crop with a farm value of over one billion U.S. dollars (American Peanut Council, 2018). Many peanut breeding programs work on developing high-yielding cultivars with resistance to biotic and abiotic stressors (e.g., drought and diseases), which may reduce the yield, quality, and the health benefits of this crop. More recently, there is an effort to utilize newly available genome sequences of domesticated peanut and its ancestral relatives (Bertioli et al., 2015) to develop improved cultivars. As a result, more work is needed on high-throughput phenotyping to connect extensive genotypic data to phenotypic characteristics in a field context (Furbank and Tester, 2011 and Fiorani and Schurr, 2013). There are numerous studies characterizing peanut germplasm for various traits, such as yield, disease resistance, and heat and drought tolerance (Holbrook and Noe, 1992; Holbrook and Anderson, 1995; Nigam et al., 2005; Bennett et al., 2018), but traditional methods for data collection are inefficient, laborious, and time-consuming for evaluating large numbers of plant genotypes.

One plant characteristic that is currently not considered in peanut breeding is canopy architecture, which has profound effects but is difficult to measure. Canopy structure affects solar radiation interception, plant growth (Suprapto et al., 2013), and ability to compete with weeds (Jannink et al., 2000). Canopy traits, such as plant size and leaf area can affect adaptation to drought conditions (Cattivelli et al., 2008). Open canopies and upright growth habits can also reduce disease incidence by creating microclimates that are less conducive to pathogen growth or by reducing opportunities for plant contact with infested soil (Shew and Beute, 1984; Dow et al., 1988; Chappell et al., 1995; Bailey and Brune, 1997). The difficulty in defining and quantifying a three-dimensional structure may be why canopy architecture is rarely measured or adopted in most crop breeding programs (Pangga et al., 2011; Tivoli et al., 2013). While there is a substantial body of literature on theoretical models of plant growth (de Visser et al., 2002; Prusinkiewicz and Runions, 2012), at a more applied level, the relatively few studies attempting to quantify aspects of canopy architecture in row crops have manually measured height, width, and/or leaf area index (Blad et al., 1978; Jannink et al., 2000; Leon et al., 2016). More recently, promising research on quantifying plant canopies use various sensors (Paulus et al., 2014; Bai et al., 2016; Hui et al., 2018), bypassing manual measurements which are low-resolution, subjective, and laborious to acquire.

Sensing technologies, such as RGB cameras, ultrasonic and infrared sensors, and laser scanners, have been used to characterize plant canopies. Image analyses are perhaps the most common method for quantifying plant parts, such as roots, stems, leaves, seeds, and flowers. Recently, 3D imaging techniques were used to describe complex geometric traits, expanding conventional 2D imaging methods with an additional dimension of distance measurement using time of flight (TOF), stereovision (Lati et al., 2013), and structure-from-motion (Li et al., 2014; Jay et al., 2015). Ultrasonic sensors were also used as a 3D sensor to evaluate canopy information in tree fruits and nuts (Escolà et al., 2011), estimate citrus yields (Zaman et al., 2006), and evaluate canopy contours of pistachio trees (Maghsoudi et al., 2015). Ultrasonic sensors are relatively inexpensive and simple to use, but their measurement accuracy is often low, being easily affected by surrounding interferences and measurement distance. Hence, ultrasonic sensors may be acceptable for evaluating fruit trees with relatively large canopies and leaves, but they are less suitable for row crops which generally have smaller canopies and smaller leaves, resulting in greater diffusion of ultrasonic waves.

Light Detection and Ranging sensors (LiDAR) are increasingly used to evaluate plant canopy architecture. This technology measures the distance between a LiDAR sensor and a targeted object based on TOF. The distance between the LiDAR and the targeted object is determined by a product of the speed of light and the time interval between when a laser signal is emitted and when the reflected laser signal is received. LiDARs were used to evaluate height, shape, structure, and contours of trees (Van der Zande et al., 2006; Côté et al., 2012; Shi et al., 2013, 2015; McMahon et al., 2015), as well as to describe surface features of vegetation canopies (Saeys et al., 2009; Li et al., 2014; Liu et al., 2017). LiDAR has the advantage of being accurate, fast, and compatible for use in outdoor environments. In addition, LiDAR is less affected by solar radiation, air temperature, humidity, or wind speed. LiDAR sensors were also used to phenotype detailed morphological features of plants. Paulus et al. (2014) built an indoor phenotyping system using a 3D, high-precision laser scanner coupled with a movable articulated arm. The system was able to scan the architecture of entire barley plants, reconstruct 3D plant models with the scanned data, estimate cumulated leaf area, height, and width, and correlate the measured features with plant growth analysis. Thapa et al. (2018) developed an indoor phenotyping system with a LiDAR sensor mounted on a rotation stage. The system generated 3D point cloud data from LiDAR-scan data, reconstructed plant leaves in digital format, and estimated morphological features of maize and sorghum leaves. Sun et al. (2017) developed a LiDAR-based high-throughput phenotyping system for cotton plants and tested the system under field conditions. An RTK GPS was used to geo-reference the LiDAR scans, and the height of every cotton plant was evaluated. Jimenez-Berni et al. (2018) designed a ground-based platform, comprised of LiDAR, digital single-lens reflex camera, and a GreenSeeker® sensor, to analyze canopy height, ground cover, and the above-ground biomass of wheat. These reports demonstrate the great potential of LiDAR technology in evaluating plant architecture indoors and in the field.

Peanut cultivars can show great variation in canopy architecture. In this study, a LiDAR sensor-based phenotyping system was developed to characterize peanut cultivars with divergent canopy architectures. The specific objectives were to: (1) design a field phenotyping platform to evaluate peanut canopy architecture; (2) to develop software to evaluate canopy features of height, width, shape, and density, and to classify among different varieties; and (3) to conduct field tests to verify the feasibility of the developed phenotyping platform.

## Materials and Methods

### System Architecture

**Figure 1:**
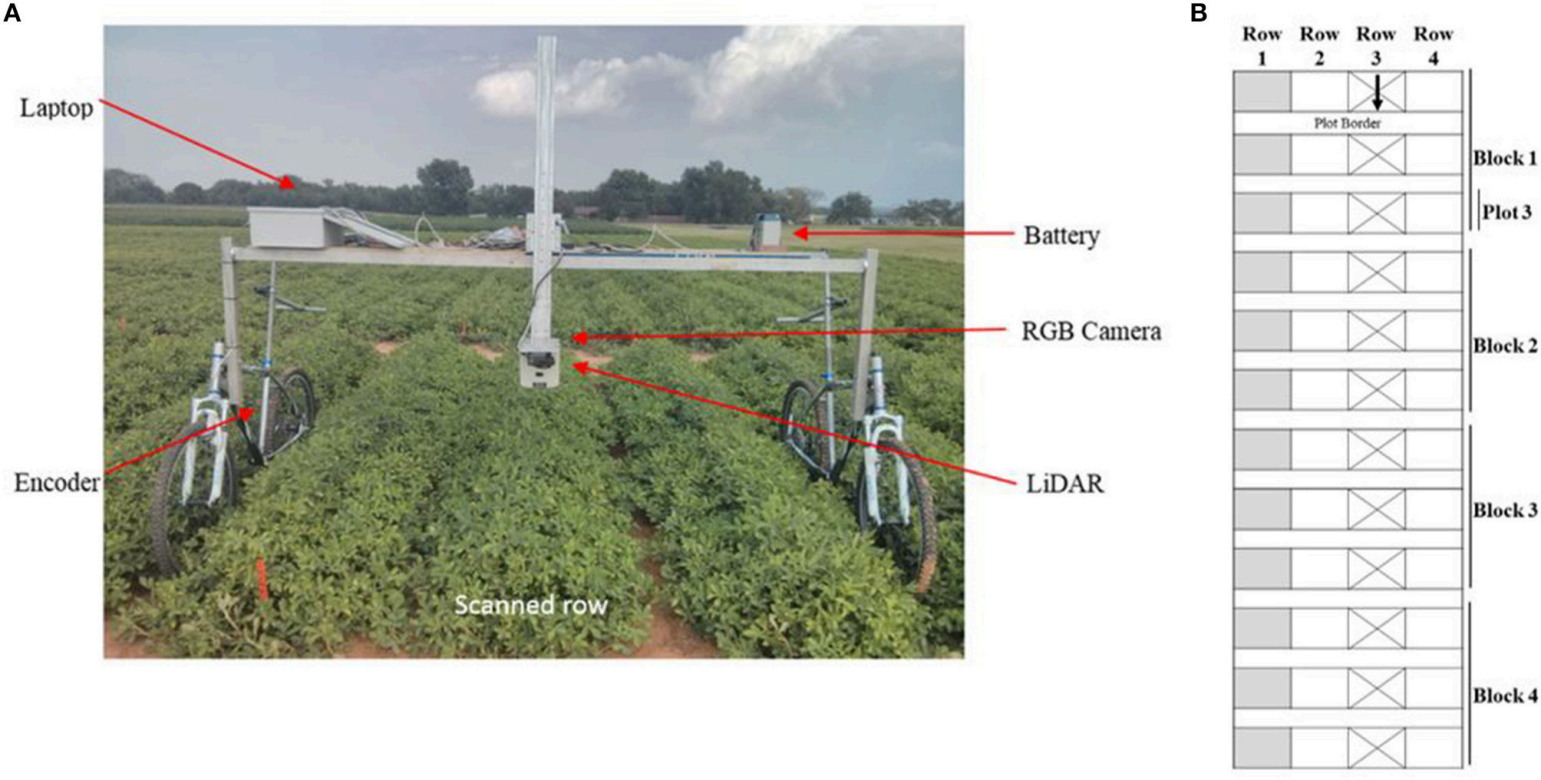
Measurement system and experimental field setup: **(A)** Ground-based mobile data acquisition system; **(B)** Experimental design of field: each plot had four rows, but only one (Row 3) was measured; Row 1 was not used. The arrow shows the travel direction of measurement system.

**Figure 2:**
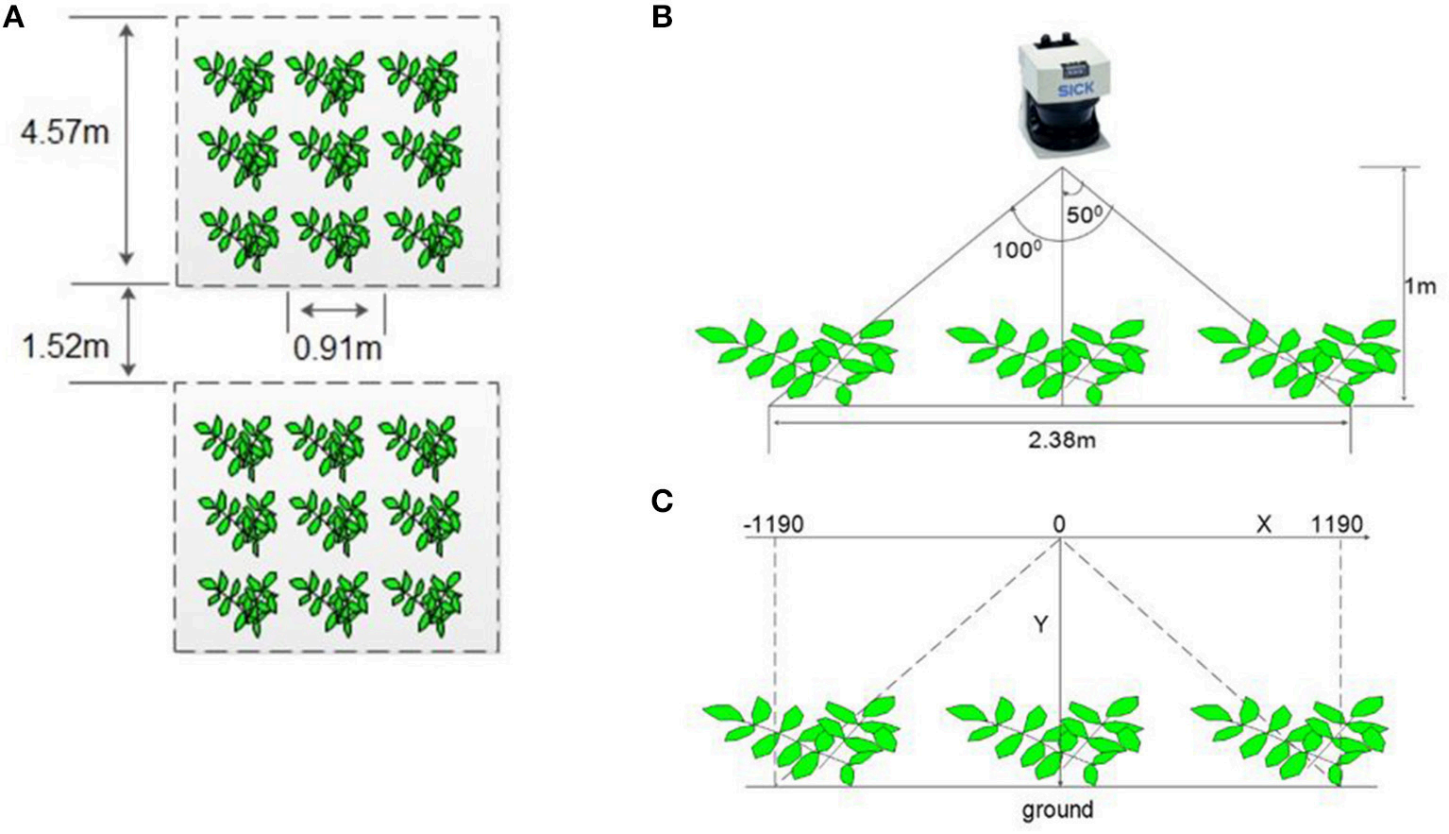
Detailed plot layout and LiDAR configuration: **(A)** The 3-row subsection of the plot covered by the field acquisition system; **(B)** Field-of-view of laser scanner; **(C)** Cartesian coordinates of the collected data.

A field phenotyping platform (Figure 1A) was constructed from two bicycles and a frame holding a ground-based LiDAR sensor (LMS291-S05, SICK AG, Waldkirch, Germany), a video camera (GoPro 4, GoPro Inc, California, USA), a shaft encoder (Danapar, Gurnee, Illinois, USA) attached to one of the rear wheels to record a location stamp of every LiDAR scan, a laptop, and a battery unit (24 V, 18.0 Amp. Hr.). The LiDAR was mounted on the platform at 1 m above ground and was oriented to face downward. To obtain a reasonable field of view of the entire canopy within a single row, the LiDAR was configured to operate in a continuous line-scan mode with a field of view of 100° (2.38 m in width) and a resolution of 0.25° (Figure 2B). The laser scanner output had a total of 401 points for every line scan. To ensure high-speed data collection (500 kbps/s), the LiDAR was connected to a laptop through a serial-to-Ethernet converter (DeviceMaster 500, Comtrol Co., New Brighton, Minnesota, USA). A data acquisition program was developed using LabVIEW (The LabVIEW 2011, National Instrument Co., Austin, Texas, USA) to communicate with the LiDAR, correctly receive data packages, extract and convert distance data from polar to Cartesian coordinates, and save data as an MS Excel file. All line-scanned data were stored with a distance stamp and a time stamp. A video camera was mounted 10 cm from the center plane of the LiDAR and oriented downward to obtain a similar field-of-view (FOV). The camera recorded videos during field tests to verify LiDAR measurements in the field.

### Field Setup and Data Acquisition

Field experiments were conducted at Oklahoma State University's Caddo Research Station in Fort Cobb, Oklahoma, USA. The following three peanut cultivars of the runner market-type were planted on June 3, 2015: Georgia-04S (Branch, 2005), McCloud (McCloud, 2006) and Southwest Runner (Kirby et al., 1998). For easier planting, each plot had four rows but only one row was measured. This plot setup permitted the designed platform to scan one row without treading on the plants within the scanned row (Figure 1). Each plot was 3.66 m in width and 4.57 m in length. The experimental design was a randomized complete block design with four replications for each cultivar. The 12 four-row-wide plots were arranged in a single line in the field (Figure 1B), and plots were separated by 1.52 m borders (Figure 2A). To provide fixed reference points within each plot over the collection periods, 0.76 m-long metal posts were installed within the center length of each scanned row at 0.9, 1.8, 2.7, and 3.7 m.

Data were collected three times during the 2015 growing season on 10 July, 21 August, and 18 September. At each collection date, the entire field of 12 plots was scanned three times with the developed mobile data acquisition system. The data from each scan were stored in separate files on the laptop.

### Ground-Truth Data Collection

The ground-truth data were obtained by taking manual measurements in each plot along the moving direction of the mobile data acquisition system at 0, 0.9, 1.8, 2.7, 3.7, and 4.6 m. At each of these six locations, canopy height was measured at seven points in the direction perpendicular to the moving direction at 15.2-cm intervals. In total, 42 ground-truth data measurements were taken in each plot, and 504 measurements for the entire field. Ground-truth data were collected on the same days that LiDAR measurements were taken.

### Software Design for Data Preprocessing

#### Raw Data

The raw data consisted of three parts: encoder data (location stamp), a time stamp, and 401 pairs of x-y coordinates of the collected data. The encoder data provided a moving distance between two LiDAR scans and was used as a location stamp. The LiDAR was configured to provide each data output as Cartesian coordinates with the center of the LiDAR as the origin. Each LiDAR scan generated 401 pairs of x-y coordinates. The x-coordinate provided horizontal displacement to the origin where a measurement was taken from, while y-coordinate provided a vertical distance measurement, which was used to evaluate the height of canopy. The horizontal scanning range, perpendicular to the row, was about −1.19 to +1.19 m (Figure 2C). When collecting field data, the LiDAR continuously acquired data with a sampling interval of 53 ms along the movement of the mobile data acquisition system.

#### Data Preprocessing

##### Plant height calculation

The plant height was calculated using Equation (1),

(1) $$\begin{array}{r} Height_{plant}=Height_{LiDAR}-y_{i},\;i=1,\;2,\;\ldots n \end{array}$$

where *n* was the total number of scanning points in direction of X-axis; for this project, *n* = 401. *Height*_*plant*_ was the calculated plant height. *Height*_*LiDAR*_ was the mounted height of the LiDAR, which was 1 m above ground level. *y*_*i*_ was the distance measured at the *ith* data point. A threshold of ±2 cm was used for *Height*_*plant*_ to represent ground.

##### Identify region of interest

**Figure 3:**
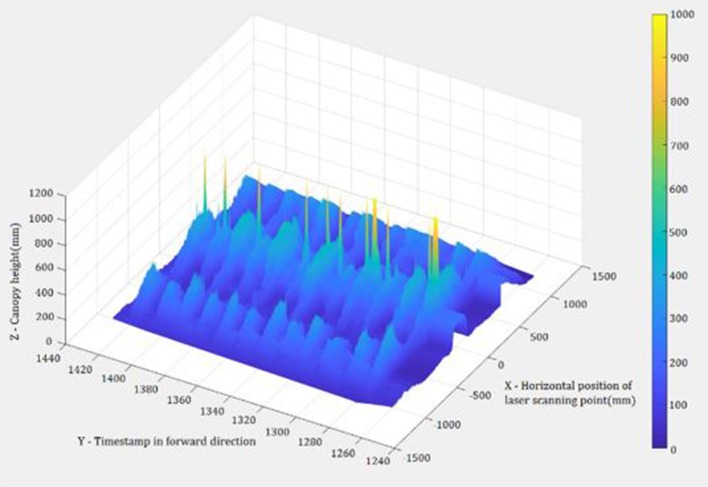
An example of the point cloud of the collected data.

A wide aperture angle of 100° was used for the LiDAR sensor to ensure a complete scan of the target canopy. As a result, the collected data included scanned canopies of adjacent rows (Figures 2, 3). A five-step algorithm was developed in MATLAB (MATLAB 2017A, MathWorks Inc., MA, USA) to extract the region of interest (ROI), i.e., the center row, through the polynomial curve fitting method:

Step 1: Separate each plot

Every data file included the collected data from all 12 plots. Equation (2) was used to extract the data for each plot based on the encoder readings.

(2) $$\begin{array}{r} Encoder_{eachplot}=\frac{\left|Encoder_{end}-Encoder_{start}\right|}{N_{plots}}, \end{array}$$

where *Encoder*_*eachplot*_ was the calculated encoder readings for each plot; Encoder_*start*_ was the encoder reading at the start of the field; Encoder_*end*_ was the encoder reading at the end of the field, and *N*_*plots*_ was the number of plots (*N*_*plots*_ = 12 in this research). As a result, the data file for a field run was divided into 12 sub-files, each representing data from the 12 plots.

Step 2: Curve fitting for the data in each sub-file

Peanut canopies were generally rounded as an arc when viewed from top (Figure 3). In order to describe canopy shape, curve fitting was implemented to fit the data from each plot. As each data file for a single plot included multiple scans, multiple fitting curves were generated using fifth-order polynomial curve fitting (Equation 3).

(3) $$\begin{array}{r} f\left(x,y\right)=\overset{n}{\underset{i=0}{\sum }}\omega_{i}x^{i},n=5, \end{array}$$

where ω_*i*_ was the coefficient of the polynomial and *n* is the order of the polynomial.

Step 3: Find the minimum and maximum points of the canopy

**Figure 4:**
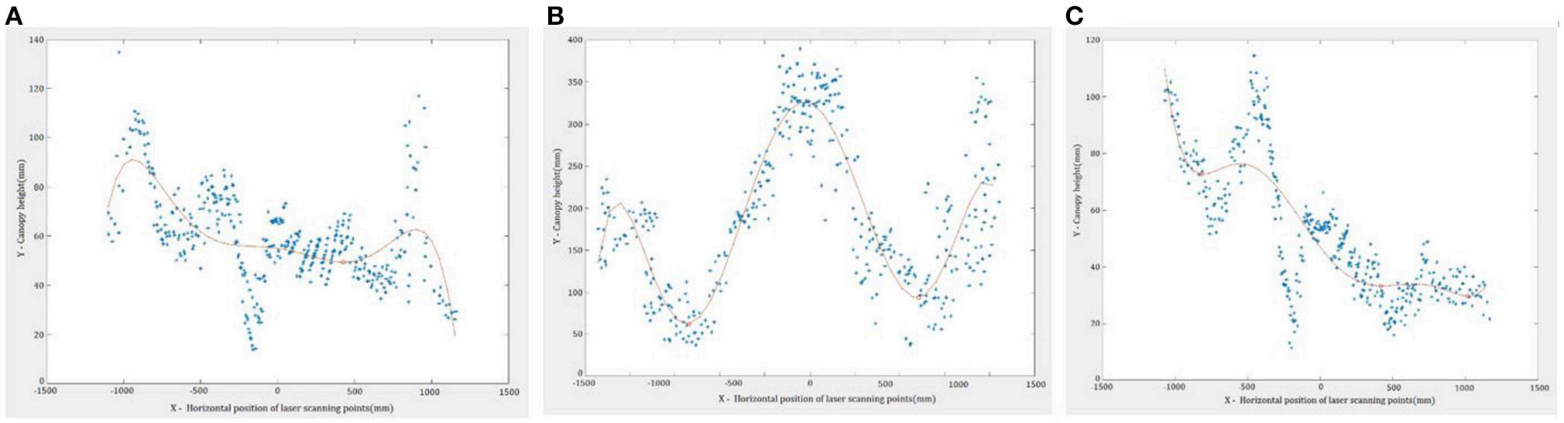
The examples of fitting curves for the data: **(A)** Distorted curve; **(B)** Curve with one peak and two valleys; **(C)** Distorted curve.

Multiple fitting curves describing canopy shape were generated in Step 2. The highest point and the left and right boundary points of the fitting curves were determined through the calculations of the maximum and minimum points in the fitting curves. Generally, the resulted curves of the peanut canopy scans from Step 2 (visible in Figures 3, 4B) have a shape with one peak (the highest height) in the middle and two valleys with minimum heights. The section of each curve between the two minimum points indicated the canopy area of interest. However, due to overlapping plants with adjacent rows, especially from data collected in August and September, some of the resulting fitted curves were distorted (Figures 4A,C). To find the targeted section in the distortion curves, the number and positions of the extreme points, especially the minimum points, of all resulted curves were evaluated.

Step 4: Determine canopy boundaries for the target plot

**Figure 5:**
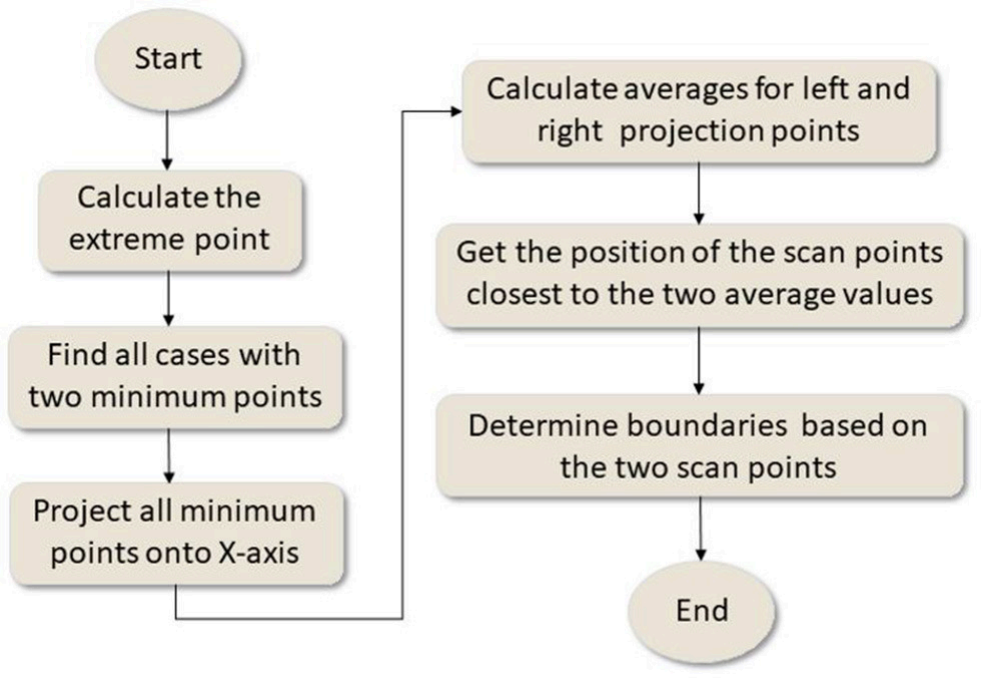
Flowchart for determining canopy boundaries.

The flowchart in Figure 5 shows the procedures to determine the boundaries of a plot.

At first, the minimum values from all fitted curves in a plot were calculated. If two minimum points (minimum-left and minimum-right) were found for a fitted curve, their x-coordinates, i.e., their positions relative to the origin, were recorded. If only one minimum point was found, the fitted curve was not used to calculate the boundaries. After finding all the pairs of the minimums for the fitted curves in a plot, the averages of the left and right minimums were calculated using Equation (4).

(4) $$\{\begin{array}{c} x_{average-left}=\frac{\sum_{i=1}^{n}x_{i}^{minimum-left}}{n}\\ x_{average-right}=\frac{\sum_{i=1}^{n}x_{i}^{minimum-right}}{n} \end{array}\;i=1,2,...n,$$

where *n* was the numbers of the fitted curves with two minimum points; *x*_*average*−*left*_ was the average x-coordinate of all the left minimum points; *x*_*average*−*right*_ was the average x-coordinate of all the right minimum points; [[Inline Image]]was the x-coordinate of the left minimum in the *ith* fitted curve; and [[Inline Image]] was the x-coordinate of the right minimum in the *ith* fitted curve.

Step 5: Extract the data in ROI

Once the two boundary coordinates *x*_*average*−*left*_and *x*_*average*−*right*_ were identified, data with x-coordinates between *x*_*averge*−*left*_ and *x*_*average*−*right*_ were considered within the ROI. The data with x-coordinates smaller than *x*_*averge*−*left*_ and larger than *x*_*average*−*right*_ were considered to be part of the plant canopies from adjacent rows and were not included in downstream processing. The identified ROI of each plot was extracted according to the canopy boundary points.

##### Remove noise and unwanted data

The presence of the four metal stakes installed in each plot were captured by LiDAR in all the data files and needed to be removed. Other additional noise was also present in the raw data. The following steps were used to remove extraneous noise and interference:

Step 1: Define a threshold operator (THO) Calculate the average height and standard deviation of each row. Next, the sum of the average height and 2 times the standard deviation was used as the judgement operator.
Step 2: Compare measurement values with THO Measurement data in a scan within a plot were retrieved and compared with THO. If the measurement value was smaller than THO, it remained unchanged and retrieved the next measurement value.
Step 3: Remove unwanted data When the measured value was greater than THO, the average of its two adjacent values (Adj_avg) was compared with THO. If THO was smaller than Adj_avg, THO was used as the measured data. If THO was greater than Adj_avg, the measurement value was replaced by Adj_avg. Return to Step 2 for the next measured value.

### Analysis for Canopy Phenotype

After preprocessing, the data file for each plot included multiple LiDAR scans. For further analyses, these multiple data scans in a plot were organized into a matrix (Equation 5).

(5) $$\begin{array}{r} plot_{i}\;={\left[\begin{array}{c} scan\;i\text{\_}1\\ scan\;i\text{\_}2\\ \vdots \\ scan\;i\text{\_}n \end{array}\right]}_{m_{i}\times n_{i}}, \end{array}$$

where *scan i_n* was the *nth* set of canopy height data within the ROI; *m*_*i*_ was the number of scans of the LiDAR in plot *i* ; and *n*_*i*_ was the number of height data in a scan within the ROI for plot *i*. The matrix (*plot*_*i*_) included all the canopy height data in the ROI. The data were processed either through standard data manipulation or as an image with image processing approaches.

#### Statistics of Peanut Canopy

Height and width are frequently used by plant scientists to describe canopy features (Dow et al., 1988; Richard et al., 2013; Hoyos-Villegas et al., 2015; Leon et al., 2016). Height and width of the three cultivars over time were analyzed using mixed-model ANOVA in PROC GLIMMIX of SAS (ver. 9.4, SAS Institute, Cary, North Carolina). Fixed effects included cultivar, month, and their interaction. Block, or repetition of cultivars, was treated as a random effect. The three scans of the field for each collection date were treated as subsamples from each plot, so the random effect of block^*^cultivar^*^month was used as the error term. Denominator degrees of freedom were corrected using the Kenward-Rogers option. When the cultivar^*^month interaction was significant, simple effects of temperatures within each week, and week within each temperature, were compared using the SLICE option of the LSMEANS statement. Type I error was controlled at α = 0.05 using the ADJUST = SIMULATE option.

#### Shape Features of Peanut Canopy

In order to obtain shape features of the peanut canopies, a MATLAB program was developed to read *plot*_*i*_as a gray-scale image. This image was converted into binary image using the threshold defined in Equation 6.

(6) $$Threshold_{i}=\frac{\sum_{b=1}^{n_{i}}\sum_{a=1}^{m_{i}}plot_{i}\left(a,b\right)|plot_{i}\left(a,b\right)>0}{n_{i}\times m_{i}},$$

If the value in *plot*_*i*_ was larger than *Threshold*_*i*_, the relevant element in the binary image was set to 1. If the value in *plot*_*i*_ was smaller than *Threshold*_*i*_, the relevant element in the binary image was set to 0. Several feature indices were calculated to describe the shape and density of the canopy (Table 1).

**Table 1:** Feature indices for canopy shape and density.

**Feature index**	**Description**
Euler number	A scalar that specified the number of holes in the region minus the objects in the region
Entropy	A scalar that characterized the aggregation characteristics of the image's gray distribution. An image with more details and larger changes in brightness, as in an open canopy, has a higher entropy value.
Cluster count	The number of connected objects found in binary image
Mean area	The mean area of the connected objects.

Canopy shape and density were described by Euler number, entropy, cluster number, and mean area of the clusters. When leaves overlap, they block the LiDAR signal from penetrating the canopy, and in the image file, overlapping leaves appeared as a cluster. If the canopy architecture was sparse and open, some LiDAR signals could pass through leaves and bounce back to the LiDAR. In the image file, these openings appeared as holes.

In plants, the Euler number approach has been used to estimate the number of flowers within a plant canopy (Adamsen et al., 2000) and the distribution of watercore symptoms in apple fruit (Melado-Herreros et al., 2013). The Euler number represents differences between the connected areas and holes in the image. A high Euler number indicates a denser canopy, with fewer holes within the canopy and relatively smooth edges in the connected regions. Entropy, representing the randomness of the gray values in an image, has been used to classify weed species (Burks et al., 2000) and detect calcium deficiency in lettuce (Story et al., 2010). Higher entropy values are obtained from images of open plant canopies due to greater changes in canopy height; images of dense canopies would have low entropy.

A cluster was defined as a group of connected objects in an image. In a binary image, each pixel (central pixel) had eight adjacent pixels, thus, the central pixel and the other eight adjacent pixels formed a 3 × 3 matrix. This matrix continued to expand if a non-zero neighboring pixel was found. The set of connected pixels formed a connected region or objects. The size and area of connected objects reflected canopy density in the image. Smaller numbers of clusters in an image indicated larger mean areas of connected regions in the image—i.e., higher density plant canopies. Sparse, open plant canopies would have larger differences in canopy height, resulting in more clusters and fewer connected objects (small mean areas).

## Results and Discussion

### Identify ROIs

**Figure 6:**
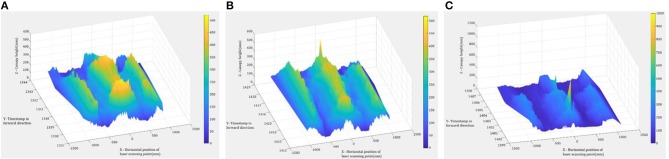
Examples of the divided sub-files: **(A)** Southwest Runner, **(B)** McCloud, and **(C)** Georgia-04S.

**Figure 7:**
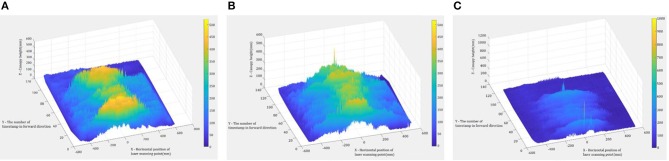
Examples of the data set with Region of Interest: **(A)** Southwest Runner, **(B)** McCloud, and **(C)** Georgia-04S.

Figure 6 shows examples of the scan data collected by the LiDAR sensor, including the target rows in the center and two adjacent rows. Figure 7 shows target rows or ROI after the neighboring rows and other unwanted data were removed. The field data set was separated into 12 plot data sets.

### Height and Width of Peanut Canopies

**Figure 8:**
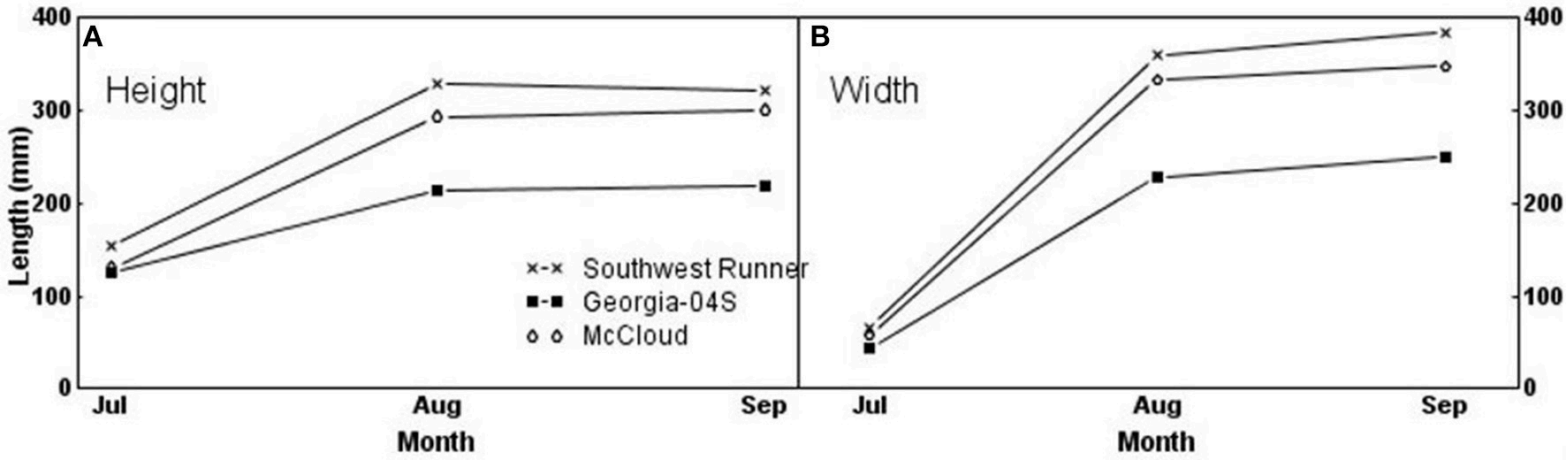
Canopy height and width of three peanut cultivars in 3 months.

Table 2 shows mean canopy height and width obtained by LiDAR for the three peanut cultivars. A significant interaction between cultivar and month was found for both height (*F* = 29.53; df = 4, 24; *P* < 0.01) and width (*F* = 28.11; df = 4, 24; *P* < 0.01), indicating that canopy height and width for each cultivar depended on month. The canopies of all cultivars grew significantly in height and width between July and August (*P* < 0.001), but growth from August to September differed only for McCloud in height and Southwest Runner in width (both *P* = 0.04). In general, Southwest Runner was the tallest and widest cultivar, and Georgia-04S had the smallest height and width (Figure 8).

**Table 2:** Mean canopy height and width of Southwest Runner, McCloud, and Georgia-04S over time^*^.

**Month**	**Cultivar**	**Height (mm)**	**Width (mm)**
July	SW Runner	154.23	64.80
	McCloud	130.03	57.59
	Georgia-04S	125.24	43.47
August	SW Runner	328.55	360.32
	McCloud	318.52	332.39
	Georgia-04S	215.01	228.46
September	SW Runner	320.69	383.59
	McCloud	298.84	348.62
	Georgia-04S	217.73	248.92

* *Standard errors: height, ± 5.5 mm; width, ± 7.5 mm*.

### Shape and Density of Peanut Canopies

Over the season, plant canopies of all cultivars generally became larger and denser (Table 3), and Euler numbers and entropy values increased over time (Tables 3, 4). However, the Euler number and entropy values for the three cultivars were very similar at the earlier growth stage in July, making it difficult to distinguish among cultivars. In later growth stages, Southwest Runner had the most open canopy, while the canopy of Georgia-04S was dense and round. Euler number appeared to be more useful than entropy for describing the changes in peanut canopy and for differentiating among the three cultivars at the later growth stages.

**Table 3:** Sample of canopy shapes and descriptors (means) for Southwest Runner, McCloud, and Georgia-04S over time.

**Month**	**Cultivar**	**Example of shape**	**Euler**	**|Entropy|**	**Cluster number**	**Mean area**
July	SW Runner	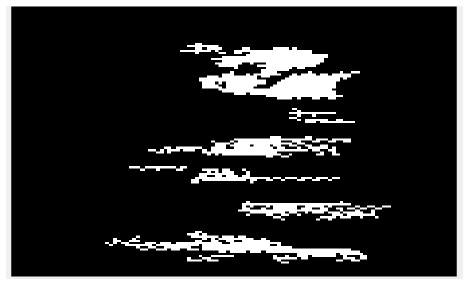	25.33	4.26	308.83	33.42
	McCloud	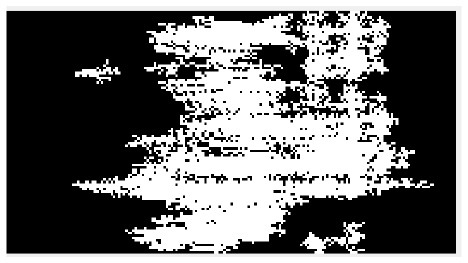	27.75	4.37	261.17	33.67
	Georgia-04S	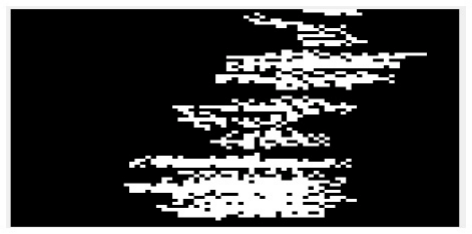	21.42	4.11	282.25	37.58
August	SW Runner	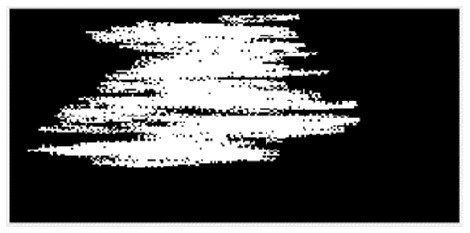	88.75	6.49	167.42	70.00
	McCloud	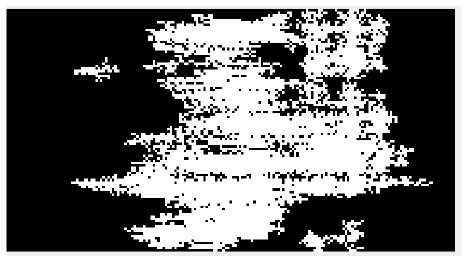	84.75	6.62	122.33	107.08
	Georgia-04S	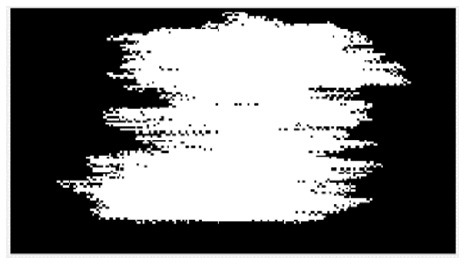	58.25	5.87	54.33	244.33
September	SW Runner	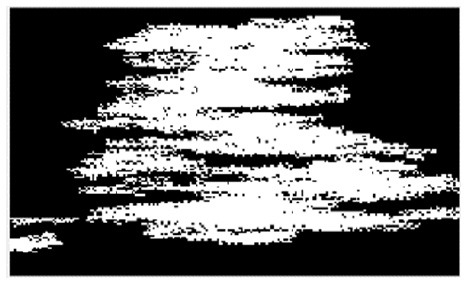	114.17	6.44	163.42	80.33
	McCloud	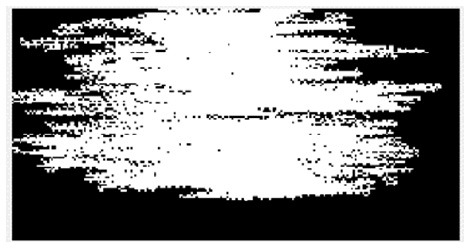	96.50	6.52	153.83	88.50
	Georgia-04S	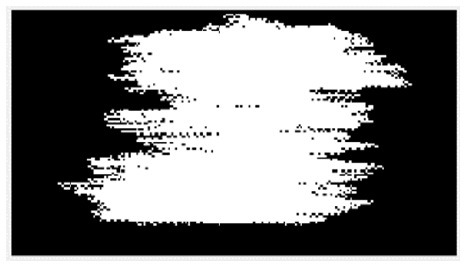	68.42	6.02	62.92	190.50

**Table 4:** Euler number and entropy estimates for canopies of Southwest Runner, McCloud, and Georgia-04S over time.^*^.

**Month**	**Cultivar**	**Euler number**				**Entropy**			
		**Mean**	**Max**	**Min**	**s.d.**	**Mean**	**Max**	**Min**	**s.d.**
July	SW Runner	25.33	44	6	10.74	4.26	4.58	4.03	0.17
	McCloud	27.75	49	4	11.86	4.37	4.64	4.09	0.18
	Geogia-S04	21.42	44	7	10.95	4.11	4.29	3.97	0.09
August	SW Runner	88.75	105	63	13.67	6.49	6.60	6.33	0.07
	McCloud	84.75	136	47	28.41	6.62	6.67	6.57	0.03
	Geogia-S04	58.25	89	31	17.51	5.87	6.09	5.59	0.16
September	SW Runner	114.17	69	185	33.58	6.44	6.55	6.31	33.58
	McCloud	96.50	72	135	21.06	6.52	6.62	6.43	21.06
	Geogia-S04	68.42	40	113	23.93	6.02	6.21	5.77	23.93

* *Values for mean, maximum, minimum, and standard deviation (s.d.) for Euler number and entropy*.

The largest number of clusters and smallest mean areas of connected objects for the three peanut cultivars occurred in July (Tables 3, 5). These numbers indicated that peanut canopies were relatively small in July and that the gaps between the leaves were relatively large. By August, the number of clusters decreased for all cultivars, and the mean area of connected objects increased relative to July. Cluster number and mean area did not change much in September from August, which was consistent with the trend for height and width. The most notable difference between August and September was a 32-point increase in cluster number and a 19-point decrease in mean area for McCloud. For the last 2 months, Georgia-04S had the fewest clusters and the largest mean area of connected objects among the three cultivars, indicating that this cultivar had the densest canopy. Canopy densities of Southwest Runner and McCloud were similar by September, but in August, canopies of McCloud were denser than those of Southwest Runner.

**Table 5:** Cluster number and mean area of connected objects for Southwest Runner, McCloud, and Georgia-04S over time.

**Month**	**Cultivar**	**Euler number**				**Entropy**			
		**Mean**	**Max**	**Min**	**s.d**.	**Mean**	**Max**	**Min**	**s.d**.
July	SW Runner	308.83	470	154	93.82	33.42	69	18	16.04
	McCloud	261.17	319	144	47.87	33.67	53	22	10.84
	Geogia-S04	282.25	475	180	81.54	37.58	46	28	6.23
August	SW Runner	167.42	217	116	34.69	70.00	132	46	25.94
	McCloud	122.33	145	103	12.46	107.08	186	55	36.42
	Geogia-S04	54.33	93	22	19.35	244.33	532	157	127.93
September	SW Runner	163.42	267	56	58.42	80.33	267	44	47.56
	McCloud	153.83	192	104	24.49	88.50	192	62	26.07
	Geogia-S04	62.92	96	46	12.59	190.50	96	123	40.58

### Comparison Between Ground-Truth Data and the Calculated Data

**Figure 9:**
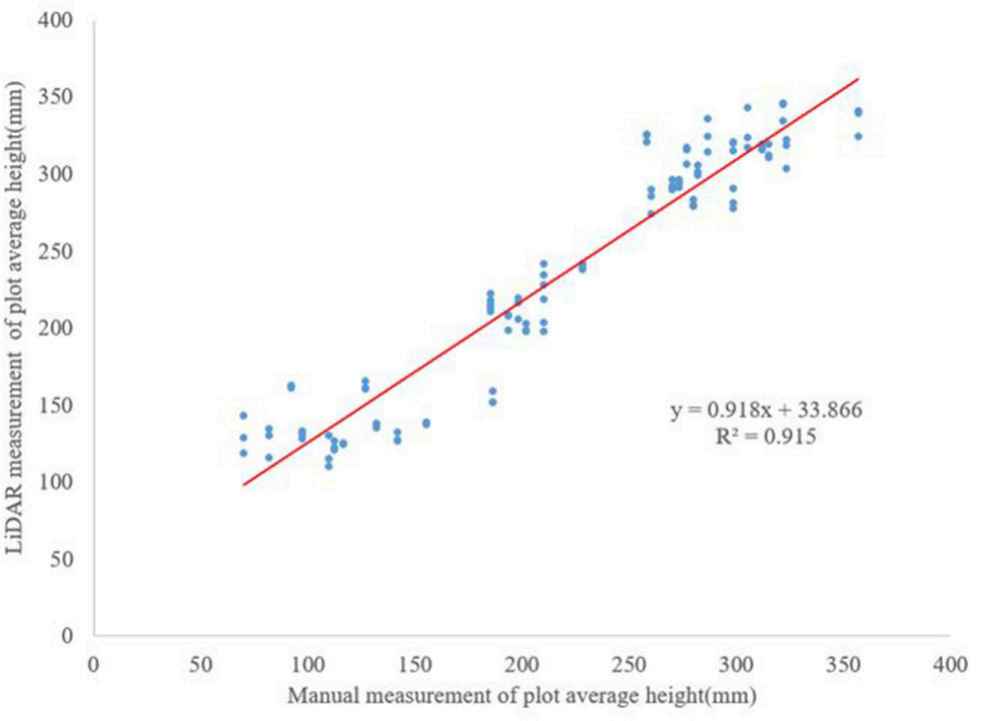
Correlation between the average heights from all plots from July to September calculated from measured data and manual ground-truth measurements.

**Figure 10:**
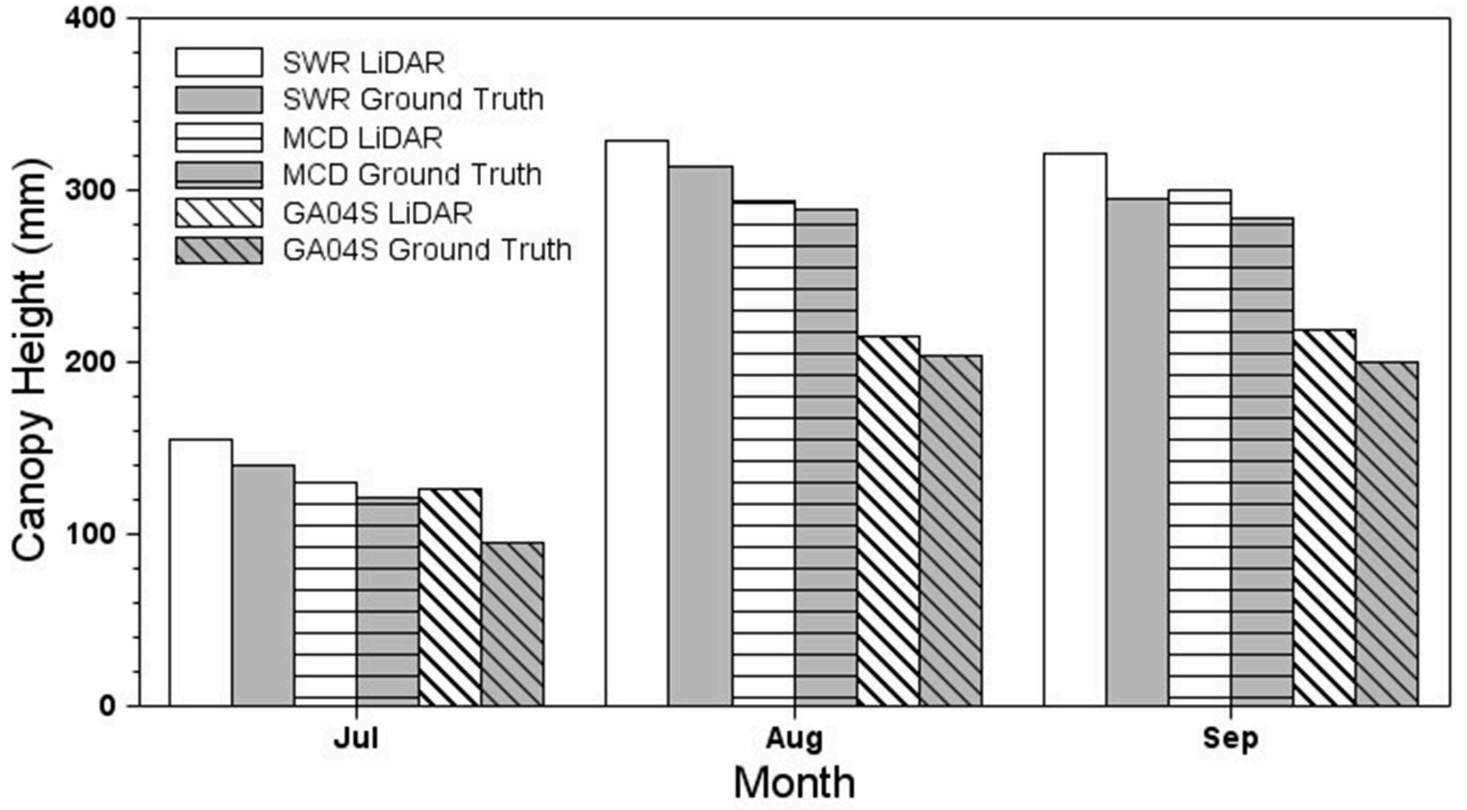
Plant heights calculated from LiDAR data vs. that from ground-truth measurements among cultivars.

There was a good correlation (*R*^2^ = 0.915; Figure 9) between the heights obtained by the developed system and manual measurements. The root-mean-squared-error was 22.78 mm. Figure 10 shows the comparison between the ground-truth measurements and the LiDAR measurement for each cultivar over time. The plant height and width measurements from LiDAR data were consistently 18.28 mm higher than the ground truth data. The error, i.e., the difference between LiDAR and ground-truth measurements, was between 5 and 24%, and the average error was 9%. Possible explanations for the error include the subjective nature of the ground-truth measurements, which were affected by wind, position of the meter stick, and human error. In addition, the vibrations of the developed mobile data acquisition system also affected LiDAR measurements. However, the LiDAR scanning platform scanned the entire field three times in the time required to collect just one set of ground-truth data from the field (504 points).

The developed LiDAR-based mobile acquisition system provided a fast and reasonably accurate way for quantifying peanut canopies. The LiDAR sensor was not affected by sun light and was easy to use and efficient in measuring canopy height compared to manual ground-truthing. However, some issues need to be considered before practical deployment: (1) Field conditions are generally not uniform, which affects the quality of the collected data. In this research, the height and width evaluations for a cultivar were evaluated by plot instead of by each LiDAR scan. Hence, resulting outliers or extreme data due to movement from platform instability were removed through statistical processing. Further study may need to be conducted to address this concern. (2) The stability of the mobile data acquisition system needs to be maintained. (3) LiDAR-based crop canopy phenotyping systems have advantages for quantifying crop canopy architecture, however they may have difficulty in differentiating various cultivars with similar architectures. For example, the methods used in this project were unable to classify the three cultivars at early growth stages when their canopy architectures were similar. In the later growth stages, when each cultivar started to show obvious differences, the developed methods were able to classify them. This system could also be used on other crops similar in height to peanut, such as alfalfa, soybean, and canola.

The complex task of breeding for a specific crop ideotype (Donald, 1968; Andrivon et al., 2012), i.e., one possessing optimal genetic and phenotypic traits, such as disease-avoiding canopy architecture, under various agronomic and environmental conditions, is difficult. However, as plant phenotyping applies more efficient technologies, such as laser scanning, 2D and 3D imaging, and spectral analyses to obtain and analyze complex phenotypic information from multiple dimensions (Khosrokhani et al., 2016; Roschera et al., 2016), breeding for ideotypes may become easier. For this project, the three peanut cultivars were chosen because they had divergent canopy shapes. Southwest Runner is highly resistant to the soilborne fungal pathogen *Sclerotinia minor* in the field, despite being susceptible when tested in a laboratory setting (Damicone et al., 2010). This cultivar's open canopy architecture may enhance disease avoidance by permitting better air circulation, solar canopy penetration, and increasing within-canopy temperatures. Without the use of the LiDAR sensor or other imaging technologies, it would have been unfeasible to collect detailed quantitative canopy characteristics for Southwest Runner and other peanut cultivars. While much work remains to determine which canopy traits (and specific quantities thereof) or combinations of traits are necessary for avoiding specific diseases, this study demonstrates the utility of LiDAR for plant canopy phenotyping.

## Conclusions

This research aimed to develop a field phenotyping system to study peanut canopy architecture. Conventional methods for in-field canopy architecture assessments require substantial investment in labor, cost, and time to acquire relatively low-resolution data. Therefore, the development of an alternative way that can rapidly measure canopy architecture is greatly desired. In this study, a ground-based LiDAR phenotyping system was developed to efficiently collect peanut canopy data in field. The results showed that LiDAR was an effective tool for assessing peanut canopy architecture under field condition. The experiment was conducted monthly in July, August and September 2015, and four replications of three cultivars were evaluated. A set of algorithms was developed to extract features of peanut canopy architecture, specifically canopy height, width, and shape/density. The descriptors used to quantify canopy shape and density, i.e., Euler number, entropy, cluster count, and mean area of connected objects, were effective for describing canopy characteristics and for discriminating among different cultivars. Finally, the canopy height data collected by LiDAR was highly correlated with ground-truth measurements with a *R*^2^ of 0.915.

## Author Contributions

HY: system design and development, data collection, and manuscript initial preparation; RB: system design, data collection, field preparation, and manuscript preparation and revision; NW: system design and development, data collection, and manuscript revision; KC: manuscript revision.

### Conflict of Interest Statement

The authors declare that the research was conducted in the absence of any commercial or financial relationships that could be construed as a potential conflict of interest. Mention of trade names or commercial products in this publication is solely for the purpose of providing specific information and does not imply recommendation or endorsement by the U.S. Department of Agriculture. USDA is an equal opportunity provider and employer.

## References

[B1] Adamsen F. J. Coffelt T. A. Nelson J. M. Barnes E. M. Rice R. C. (2000). Method for using images from a color digital camera to estimate flower noumber. Crop Sci. 40, 704–709. 10.2135/cropsci2000.403704x

[B2] American Peanut Council (2018). American Peanut Council U.S. Peanut Supply. Available online at: https://www.peanutsusa.com/about-peanuts/the-peanut-industry3/18-u-s-peanut-supply.html (accessed September 17, 2018).

[B3] Andrivon D. Giorgetti C. Baranger A. Calonnec A. Cartolaro P. Faivre R. (2012). Defining and designing plant architectural ideotypes to control epidemics? Eur. J. Plant Pathol. 135, 611–617. 10.1007/s10658-012-0126-y

[B4] Bai G. Ge Y. Hussain W. Baenziger P. S. Graef G. (2016). A multi-sensor system for high throughput field phenotyping in soybean and wheat breeding. Comput. Electron. Agric. 128, 181–192. 10.1016/j.compag.2016.08.021

[B5] Bailey J. E. Brune P. D. (1997). Effect of crop pruning on Sclerotinia blight of peanut. Plant Dis. 81, 990–995. 10.1094/PDIS.1997.81.9.990 30861984

[B6] Bennett R. S. Chamberlin K. D. Damicone J. P. (2018). Sclerotinia blight resistance in the US peanut mini-core collection. Crop Sci. 58, 1306–1317. 10.2135/cropsci2017.09.0591

[B7] Bertioli D. J. Cannon S. B. Froenicke L. Huang G. Farmer A. D. Cannon E. K.. (2015). The genome sequences of Arachis duranensis and Arachis ipaensis, the diploid ancestors of cultivated peanut. Nat. Genet. 48, 438–446. 10.1038/ng.3517 26901068

[B8] Blad B. L. Steadman J. L. Weiss A. (1978). Canopy structure and irrigation influence white mold disease and microclimate of dry edible beans. Phytopathology 68, 1431–1437. 10.1094/Phyto-68-1431

[B9] Branch W. D. (2005). Registration of ‘Georgia-04S’ peanut. Crop Sci. 45, 1653–1654. 10.2135/cropsci2004-059

[B10] Burks T. F. Shearer S. A. Payne F. A. (2000). Classification of weed species using color texture features and discriminant analysis. Trans. ASAE 43:441 10.13031/2013.2723

[B11] Côté J. F. Fournier R. A. Frazer G. W. Niemann K. O. (2012). A fine-scale architectural model of trees to enhance LiDAR-derived measurements of forest canopy structure. Agric. Meteorol. 166, 72–85. 10.1016/j.agrformet.2012.06.007

[B12] Cattivelli L. Rizza F. Badeck F. W. Mazzucotelli E. Mastrangelo A. M. Francia E. (2008). Drought tolerance improvement in crop plants: an integrated view from breeding to genomics. Field Crops Res. 105, 1–14. 10.1016/j.fcr.2007.07.004

[B13] Chappell G. F. Shew B. B. Ferguson J. M. Beute M. K. (1995). Mechanisms of resistance to *Sclerotinia minor* in selected peanut genotypes. Crop Sci. 35, 692–696. 10.2135/cropsci1995.0011183X003500030007x

[B14] Damicone J. P. Holbrook C. C. Smith D. L. Melouk H. A. Chamberlin K. D. (2010). Reaction of the core collection of peanut germplasm to Sclerotinia blight and pepper spot. Peanut Sci. 37, 1–11. 10.3146/PS09-001.1

[B15] de Visser P. H. B. Marcelis L. F. M. van der Heijden G. W. A. M. Vos J. Stuick P. Evers J. (2002). 3D Modeling of Plants: A Review. Technical Report Wageningen. Plant Research International B.

[B16] Donald C. M. (1968). The breeding of crop ideotypes. Euphytica 17, 385–403. 10.1007/BF00056241

[B17] Dow R. L. Powell N. L. Porter D. M. (1988). Effects of modification of the plant canopy environment on Sclerotinia blight of peanut. Peanut Sci. 15, 1–5. 10.3146/i0095-3679-15-1-1

[B18] Escolà A. Planas S. Rosell J. R. Pomar J. Camp F. Solanelles F.. (2011). Performance of an ultrasonic ranging sensor in apple tree canopies. Sensors 11, 2459–2477. 10.3390/s110302459 22163749 PMC3231637

[B19] Fiorani F. Schurr U. (2013). Future scenarios for plant phenotyping. Annu. Rev. Plant Biol. 64, 267–291. 10.1146/annurev-arplant-050312-120137 23451789

[B20] Furbank R. T. Tester M. (2011). Phenomics – technologies to relieve the phenotyping bottleneck. Trends Plant Sci. 16, 635–644. 10.1016/j.tplants.2011.09.005 22074787

[B21] Holbrook C. C. Anderson W. F. (1995). Evaluation of a core collection to identify resistance to late leafspot in peanut. Crop Sci. 35, 1700–1702. 10.2135/cropsci1995.0011183X003500060032x

[B22] Holbrook C. C. Noe J. P. (1992). Resistance to the peanut root-knot nematode (*Meloidogyne arenaria*) in *Arachis hypogaea*. Peanut Sci. 19, 35–37. 10.3146/i0095-3679-19-1-9

[B23] Hoyos-Villegas V. Mkwaila W. Cregan P. B. Kelly J. D. (2015). Quantitative trait loci analysis of white mold avoidance in pinto bean. Crop Sci. 55, 2116–2129. 10.2135/cropsci2015.02.0106

[B24] Hui F. Zhu J. Hu P. Meng L. Zhu B. Guo Y. (2018). Image-based dynamic quantification and high-accuracy 3D evaluation of canopy structure of plant populations. Ann. Bot. 121, 1079–1088. 10.1093/aob/mcy016 29509841 PMC5906925

[B25] Jannink J.-L. Orf J. H. Jordan N. R. Shaw R. G. (2000). Index selection for weed suppressive ability in soybean. Crop Sci. 40, 1087–1094. 10.2135/cropsci2000.4041087x

[B26] Jay S. Rabatel G. Hadoux X. Moura D. Gorretta N. (2015). In-field crop row phenotyping from 3D modeling performed using structure from motion. Comput. Electron. Agric. 110, 70–77. 10.1016/j.compag.2014.09.021

[B27] Jimenez-Berni J. Deery D. M. Rozas-Larraondo P. Condon A. T. G. Rebetzke G. J. James R. A.. (2018). High throughput determination of plant height, ground cover, and above-ground biomass in wheat with LiDAR. Front. Plant. Sci. 9:237. 10.3389/fpls.2018.00237 29535749 PMC5835033

[B28] Khosrokhani M. Khairunniza-Bejo S. Pradhan B. (2016). Geospatial technologies for detection and monitoring of Ganoderma basal stem rot infection in oil palm plantations: a review on sensors and techniques. Geocarto Int. 2016, 1–17. 10.1080/10106049.2016.1243410

[B29] Kirby J. S. Melouk H. A. Stevens T. E. Banks D. J. Sholar J. R. Damicone J. P. (1998). Registration of ‘southwest runner’ peanut. Crop Sci. 38, 545–546. 10.2135/cropsci1998.0011183X003800020065x

[B30] Lati R. N. Filin S. Eizenberg H. (2013). Estimating plant growth parameters using an energy minimization-based stereovision model. Comput. Electron. Agric. 98, 260–271. 10.1016/j.compag.2013.07.012

[B31] Leon R. G. Mulvaney M. J. Tillman B. L. (2016). Peanut cultivars differing in growth habit and canopy architecture respond similarly to weed interference. Peanut Sci. 43, 133–140. 10.3146/PS16-3.1

[B32] Li L. Zhang Q. Huang D. (2014). A review of imaging techniques for plant phenotyping. Sensors 14, 20078–20111. 10.3390/s141120078 25347588 PMC4279472

[B33] Liu S. Baret F. Abichou M. Bouden F. Thomas S. Zhao K. (2017). Estimating wheat green area index from ground-based LiDAR measurement using a 3D canopy structure model. Agric. Meteorol. 247, 12–20. 10.1016/j.agrformet.2017.07.007

[B34] Maghsoudi H. Minaei S. Ghobadian B. Masoudi H. (2015). Ultrasonic sensing of pistachio canopy for low-volume precision spraying. Comput. Electron. Agric. 112, 149–160. 10.1016/j.compag.2014.12.015

[B35] McCloud (2006). Peanut. University of Florida. U.S. Plant Variety Protection No. 200800232.

[B36] McMahon S. M. Bebber D. P. Butt N. Crockatt M. Kirby K. Parker G. G. (2015). Ground based LiDAR demonstrates the legacy of management history to canopy structure and composition across a fragmented temperate woodland. For. Ecol. Manag. 335, 255–260. 10.1016/j.foreco.2014.08.039

[B37] Melado-Herreros A. Muñoz-García M.-A. Blanco A. Val J. Fernández-Valle M. E. Barreiro P. (2013). Assessment of watercore development in apples with MRI: Effect of fruit location in the canopy. Postharvest Biol. Technol. 86, 125–133. 10.1016/j.postharvbio.2013.06.030

[B38] Nigam S. N. Chandra S. Sridevi K. R. Bhukta M. Reddy A. G. S. Rachaputi N. R. (2005). Efficiency of physiological trait-based and empirical selection approaches for drought tolerance in groundnut. Ann. Appl. Biol. 146, 433–439. 10.1111/j.1744-7348.2005.040076.x

[B39] Pangga I. B. Hannan J. Chakraborty S. (2011). Pathogen dynamics in a crop canopy and their evolution under changing climate. Plant Pathol. 60, 70–81. 10.1111/j.1365-3059.2010.02408.x

[B40] Paulus P. Schumann H.:, Kuhlmann, H. Leon J. (2014). High-precision laser scanning system for capturing 3D plant architecture and analysing growth of cereal plants. Biosyst. Eng. 121, 1–11. 10.1016/j.biosystemseng.2014.01.010

[B41] Prusinkiewicz P. Runions A. (2012). Computational models of plant development and form. New Phytol. 193, 549–569. 10.1111/j.1469-8137.2011.04009.x 22235985

[B42] Richard B. Bussière F. Langrume C. Rouault F. Jumel S. Faivre R. (2013). Effect of pea canopy architecture on microclimate and consequences on ascochyta blight infection under field conditions. Eur. J. Plant Pathol. 135, 509–524. 10.1007/s10658-012-0132-0

[B43] Roschera R. Behmanna J. Mahlein A.-K. Dupuis J. Kuhlmann H. Plumer L. (2016). Detection of disease sympotoms on hyperspectral 3D plant models. ISPRS Ann. Photogramm. Rem. Sens. Spatial. Inform. Sci. 3, 89–96. 10.5194/isprsannals-III-7-89-2016

[B44] Saeys W. Lenaerts B. Craessaerts G. De Baerdemaeker J. (2009). Estimation of the crop density of small grains using LiDAR sensors. Biosyst. Eng. 102, 22–30. 10.1016/j.biosystemseng.2008.10.003

[B45] Shew B. B. Beute M. K. (1984). Effects of crop management on the epidemiology of southern stem rot of peanut. Phytopathology 74, 530–535. 10.1094/Phyto-74-530

[B46] Shi Y. Wang N. Taylor R. K. Raun W. R. (2015). Improvement of a Ground-LiDAR-based corn plant population and spacing measurement system. Comput. Electron. Agric. 112, 92–101. 10.1016/j.compag.2014.11.026

[B47] Shi Y. Wang N. Taylor R. K. Raun W. R. Hardin J. A. (2013). Automatic corn plant location and spacing measurement using laser line-scan technique. J. Precision Agric. 4, 478–494. 10.1007/s11119-013-9311-z

[B48] Story D. Kacira M. Kubota C. Akoglu A. An L. (2010). Lettuce calcium deficiency detection with machine vision computed plant features in controlled environments. Comput. Electron. Agric. 74, 238–243. 10.1016/j.compag.2010.08.010

[B49] Sun S. Li C. Paterson A. H. (2017). In-field high-throughput phenotyping of cotton plant height using LiDAR. Remote Sens. 9:377 10.3390/rs9040377 PMC5786533 29403522

[B50] Suprapto A. Sugito Y. Sitompul S. M. (2013). Study of growth, yield and radiation energy conversion efficiency on varieties and different plant population of peanut. Procedia Environ. Sci. 17, 37–45. 10.1016/j.proenv.2013.02.009

[B51] Thapa S. Zhu F. Walia H. Yu H. Ge Y. (2018). A novel LiDAR-based instrument for high-throughput, 3D measurement of morphological traits in maize and sorghum. Sensors 18:1187. 10.3390/s18041187 29652788 PMC5948551

[B52] Tivoli B. Calonnec A. Richard B. Ney B. Andrivon D. (2013). Current knowledge on plant/canopy architectural traits that reduce the expression and development of epidemics. Eur. J. Plant Pathol. 135, 471–478. 10.1007/s10658-012-0066-6

[B53] Van der Zande D. Hoet W. Jonckheere I. van Aardt J. Coppin P. (2006). Influence of measurement set-up of ground-based LiDAR for derivation of tree structure. Agric. For. Meteorol. 141, 147–160. 10.1016/j.agrformet.2006.09.007

[B54] Zaman Q. U. Schumann A. W. Hostler H. K. (2006). Estimation of citrus fruit yield using ultrasonically-sensed tree size. Appl. Eng. Agric. 22, 39–44. 10.13031/2013.20186

